# A Catalogue of Putative *cis*-Regulatory Interactions Between Long Non-coding RNAs and Proximal Coding Genes Based on Correlative Analysis Across Diverse Human Tumors

**DOI:** 10.1534/g3.118.200296

**Published:** 2018-04-17

**Authors:** Swaraj Basu, Erik Larsson

**Affiliations:** Department of Medical Biochemistry and Cell Biology, Institute of Biomedicine, The Sahlgrenska Academy, University of Gothenburg, SE-405 30 Gothenburg, Sweden

**Keywords:** cancer, RNAseq, lncRNA, transcriptome, correlation

## Abstract

Antisense transcripts and other long non-coding RNAs are pervasive in mammalian cells, and some of these molecules have been proposed to regulate proximal protein-coding genes in *cis*. For example, non-coding transcription can contribute to inactivation of tumor suppressor genes in cancer, and antisense transcripts have been implicated in the epigenetic inactivation of imprinted genes. However, our knowledge is still limited and more such regulatory interactions likely await discovery. Here, we make use of available gene expression data from a large compendium of human tumors to generate hypotheses regarding non-coding-to-coding *cis*-regulatory relationships with emphasis on negative associations, as these are less likely to arise for reasons other than *cis*-regulation. We document a large number of possible regulatory interactions, including 193 coding/non-coding pairs that show expression patterns compatible with negative *cis*-regulation. Importantly, by this approach we capture several known cases, and many of the involved coding genes have known roles in cancer. Our study provides a large catalog of putative non-coding/coding *cis*-regulatory pairs that may serve as a basis for further experimental validation and characterization.

Long non-coding RNAs (lncRNAs) are implicated in vital cellular processes like chromatin modification, regulation of transcription and molecular scaffolding (Quinn and Chang 2016). Several lncRNAs are reported to act in a *cis*-regulatory fashion, modulating the transcription of nearby genes using diverse mechanisms, as shown for lncRNAs like *MALAT1* (Zhang *et al.*, 2012), *HOTTIP* (Burgess 2011), and *ANRIL* (Yap *et al.*, 2010). Non-coding RNAs overlapping coding genes in the antisense orientation can also regulate their coding partners, often in an inhibitory fashion, as proposed for the tumor suppressor *P15* and its antisense RNA *P15AS* in mouse (Yu *et al.*, 2008). Similarly, allele-specific repression of several imprinted genes has been shown to involve non-coding RNA transcription, including the lncRNA *UBE3A-ATS* in humans, proposed to cause repression of the antisense overlapping coding gene *UBE3A* through transcriptional collision (Meng *et al.*, 2012), and *KCNQ1OT1*, which is involved in epigenetic silencing of its antisense partner *KCNQ1* and multiple other coding genes in *cis KCNQ1OT1*(Du *et al.*, 2004). These examples, and a relatively limited number of other well-characterized cases, are likely to only represent a small subset all *cis*-regulatory interactions involving non-coding RNAs and proximal coding genes.

An appealing idea is to make use of the abundance of expression data available in the public domain to make predictions about lncRNA *cis*-regulatory interactions. A recent report based on several hundred tumor samples showed widespread transcription of antisense lncRNAs, which were mostly positively correlated with their proximal coding partners, and identified potential antisense molecules that may be involved in the regulation of oncogenes and tumor suppressors (Balbin *et al.*, 2015). Likewise, earlier reports have also described a general trend of positive expression correlation for coding/lncRNAs proximal pairs across diverse samples, in particular for antisense overlapping transcripts (Derrien *et al.*, 2012). In general, these positive associations are likely to reflect shared chromatin domains or transcriptional ripple effects rather than a direct regulatory interaction (Le Dily *et al.*, 2014), and the same trend is indeed observed also for closely spaced protein-coding genes (Ghanbarian and Hurst 2015), thereby making it difficult to discern signal from noise. Negative correlations, in contrast, are not as easily explained by such trivial effects. One may therefore argue that it may be more viable to search for inhibitory *cis*-regulatory interactions, as described for some antisense transcripts, with the help of correlative gene expression analyses.

Here, we aimed to generate a catalog of putative *cis*-regulatory interactions between lncRNAs and nearby protein-coding genes, based on an analysis of transcriptome sequencing data from over 9000 tumor samples across 32 cancer types from The Cancer Genome Atlas consortium. By quantifying the expression of ∼20,000 coding genes and ∼13,000 lncRNAs we were able to identify a subset of lncRNAs that may potentially act as repressors of proximal coding genes in one or more cancer types, as indicated by patterns of negative expression correlation across tumor samples. These results constitute a powerful resource that may serve as a starting point for experimental studies in search of biologically active non-coding genes, which may in the extension serve as biomarkers or therapeutic targets in cancer.

## Materials and Methods

The raw sequencing data download and analysis was performed as described in a previous study (Ashouri *et al.*, 2016). In brief, raw sequencing reads were downloaded from https://cghub.ucsc.edu/ using the gene torrent client (https://annaisystems.zendesk.com) for 10422 samples falling under 33 cancer types. The reads were mapped on the human genome (hg19) using the hisat aligner v0.1.6-beta (Kim *et al.*, 2015) where parameters were chosen to ensure filtering of mixed and discordant reads along with better accuracy of alignment at splice sites (–no-mixed–no-discordant–no-unal –known-splicesite-infile). The gene quantification step was performed by HTSeq v0.6.0 (Anders *et al.*, 2015) ignoring reads overlapping multiple genomic features (-m intersection-strict). The gene wise raw read counts obtained from HTSeq (GENCODE v19 annotation) for primary tumor samples (9153 samples from 32 cancer types) were further normalized by library size to obtain expression metric as counts per million using the edgeR package v3.6.8 in the R statistical environment (Robinson *et al.*, 2010). For each cancer type, genes showing an expression of at least 1 cpm in 3 or more samples were retained for downstream analyses. The data.table package in R was used extensively to handle large expression matrices (https://CRAN.R-project.org/package=data.table).

The BEDTools software suite v2.25 (Quinlan and Hall 2010) was used to identify lncRNA classes based on their position with respect to their proximal coding genes. In brief, windowBed binary was used to define a flanking region of 25 kb, 50 kb and 100 kb for each coding gene and identify the lncRNAs and other coding genes falling within the specified boundary. Further intersectBed and closestBed binary were used to identify all lncRNAs transcribed in sense within 5 kb distance of a coding gene. These lncRNAs were marked as potential alternative polyadenylation events and not considered for the downstream analysis. The rest, based on the distance threshold were placed in coding/coding or coding/lncRNAs pairs at 25 kb, 50 kb and 100 kb. Finally using the shuffleBed (-chrom) utility coding genes and lncRNA genomic coordinates were shuffled and then windowBed was used to identify coding/lncRNA pairs falling within 25 kb, 50 kb and 100 kb distance of each other to generate a randomized dataset. Correlation of expression was measured between the identified coding/lncRNA and coding/coding pairs along with the shuffled pairs using spearman correlation coefficient (*ρ*) across all samples for each given cancer type. Correlations are deemed to be significant if the corrected *P* (Benjamini & Hochberg) was <1e-5. Since several cancer types contain a large number of samples we refrained from filtering the results on the estimated spearman correlation coefficient while keeping a stringent *P* cut-off. Gene expression is also dependent on somatic mutations and copy number variations in cancer genomes, thus avoiding a threshold for *ρ* allowed us to consider gene pairs with a lower expression correlation which may have biological significance.

The mean cpm of each gene across all samples in a given cancer type was used to estimate the *Tau* expression specificity score as described in a recent review (Kryuchkova-Mostacci and Robinson-Rechavi 2017). The sequence conservation for coding genes was measured by averaging the phastcons score over all exons of each coding genes using the bigWigAverageOverBed binary from UCSC utilities and the groupBy binary from BEDTools. The phastcons 100way conservation data in bigwig format was downloaded from the UCSC database (http://hgdownload.cse.ucsc.edu/goldenpath/hg19/phastCons100way/hg19.100way.phastCons.bw). A similar approach was used to measure the sequence conservation of lncRNAs except lncRNA exonic regions which overlap with coding exons were removed using the substractBed utility from BEDTools. The methylation data across 32 cancer types (Human Methylation 450 platform) was downloaded from TCGA data portal with the bioconductor package TCGAbiolinks (Colaprico *et al.*, 2015). The methylation probes were associated with gene transcription start site (TSS) or body using an annotation file provided by Illumina (ftp://webdata2:webdata2@ussd-ftp.illumina.com/downloads/ProductFiles/HumanMethylation450/HumanMethylation450_15017482_v1-2.csv). If multiple probes matched a given TSS, the probe with the maximum value was considered to be representative of the TSS. Spearman correlation coefficient was estimated between the methylation beta values at coding gene TSS and lncRNA gene expression for matched samples within each cancer type. A corrected *P* cut-off of 0.05 was used to select significant correlations.

### Data Availability

The authors affirm that all data necessary for confirming the conclusions of the article are present within the article, figures, and tables. Supplemental material available at Figshare: https://doi.org/10.25387/g3.6106808.

## Results and Discussion

### Quantification of lncRNAs and coding genes across thousands of tumors

**Figure 1:**
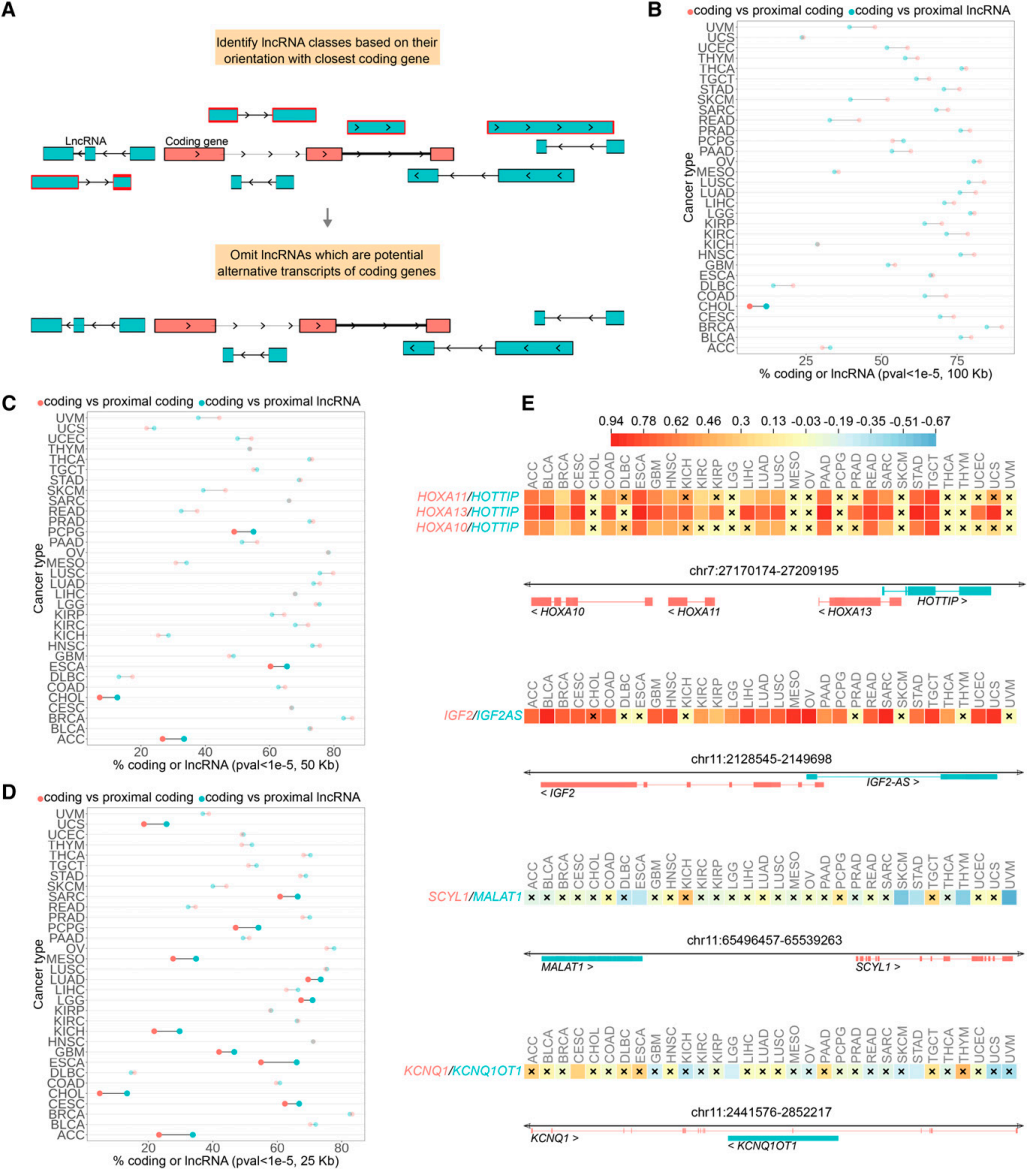
General statistics of coding/coding and lncRNAs/coding correlations at several genomic distance thresholds along with specific examples. A) Classification of lncRNAs based on their orientation with respect to a flanking coding gene. LncRNAs marked in red might be potential alternative polyadenylation events of coding genes and are hence removed from downstream analyses. B-D) Enrichment of lncRNAs for significant correlations with proximal coding genes at different genomic distance thresholds: B) 100 Kb C) 50 Kb D) 25 Kb. Cancer types where the fraction of lncRNA-coding proximal pairs that show significant correlation is significantly enriched compared to control sets of coding-coding pairs are indicated by larger dots (Fisher’s exact test, *P* < 0.01). E) Examples of known lncRNAs-coding regulatory interactions that are also confirmed by the correlative analysis. Each box represents the estimated spearman correlation coefficient between the coding gene and the lncRNAs in a given cancer type. The colors correspond to the values depicted in the scale. Crosses indicate correlations at *P* > 1e-5.

Raw RNA sequencing data from 9,193 primary tumor samples across 32 cancer types were downloaded and mapped to the human genome to quantify 60,296 coding and non-coding gene features in the GENCODE (v19) annotation (Figure S1a). Since lncRNAs are significantly enriched for repeats like transposable elements (Kelley and Rinn 2012) we excluded multi-mapping reads and discordant read pairs to reduce erroneous quantification. As an added measure we omitted mapped reads overlapping multiple genomic features (both coding and lncRNA exons) prior to the quantification of genomic features (Figure S1b). These stringent filtering measures reduce the probability of detecting false positive correlations between coding genes and lncRNAs. Additionally we filtered out lncRNAs that were possible alternative polyadenylation events of coding genes (Hoque *et al.*, 2013) (Figure 1a, see Material and methods).

### General patterns of correlation between coding genes and proximal lncRNAs

Genes that reside in the same genomic neighborhood often tend to be co-expressed, and this effect has been shown to be pronounced within a distance threshold of 100 kb in the human genome (Ghanbarian and Hurst 2015; Thygesen and Zwinderman 2005). Taking these previous reports into account, first we calculated the spearman correlation coefficient (ρ) between coding genes and all lncRNAs overlapping completely or partially with a window extending +/− 100 kb from the boundaries of each coding gene. As expected, the ρ distribution for proximal coding/lncRNA pairs was skewed toward positive (Figure S2) as compared against random coding/lncRNA pairs (Figure S3), in all cancer types analyzed. We also observed that the fraction of lncRNAs whose expression significantly correlates (+/− ρ) with their neighboring coding genes (*P* < 1e-5) was higher than the random expectation (Figure S4 a, b; Fishers exact test, *P* < 0.01). These results are consistent with earlier reports, and are likely to largely reflect shared transcriptional programs of co-localized genes residing in common topological domains (Le Dily *et al.*, 2014), rather than positive regulation of coding genes by lncRNAs in *cis*.

To normalize for the effect of coordinated gene expression within genomic blocks, we next compared the expression of each coding gene against neighboring lncRNAs at different distance thresholds (100 kb, 50 kb and 25 kb), considering each cancer type independently. Here, 64%, 57% and 50% of the total lncRNA set had a coding gene within 100 kb, 50 kb and 25 kb, respectively. As a control, the same analysis was also done while instead comparing against neighboring coding genes within the same distance thresholds. Finally, we compared the fraction lncRNAs/coding pairs showing significant correlation (positive or negative ρ, *P <* 1e-5) to the control (coding/coding) pairs. This revealed that the lncRNAs/coding pairs were enriched in terms of significant correlations compared to coding/coding pairs at shorter (<25 kb) genomic distances (Figure 1b-d). Expectedly, the vast majority of lncRNA/coding correlations were positive, with a higher fraction of positive correlations found at shorter distances (84.9% at 25 kb compared to 79.6% at 100 kb; Figure S5). LncRNAs thus tend to be associated at the gene expression level with their proximal coding genes, to a larger extent than similar proximal coding/coding pairs. While this could in part be an outcome of *cis*-regulatory functions, in most cases it is more likely a reflection of lncRNAs relying to a larger extent on the transcription of their neighboring coding genes for their activation, due to the absence of an independent transcriptional module.

### Expected positive and negative correlations for several known cis acting lncRNAs

When compared against a database of experimentally validated lncRNAs (Quek *et al.*, 2015) we found that the results of the correlation analysis supported several established regulatory interactions (Table S1). The lncRNA *HOTTIP* showed strong positive correlation with *HOXA* genes, consistent with its described role in activating *HOX* cluster genes (Wang *et al.*, 2011) (Figure 1e). However, it must again be noted that such positive correlations provide limited evidence for regulatory interactions. As an example, while the lncRNA *IGF2AS* showed strong positive correlation with its host coding gene *IGF2* gene across several cancer types (Figure 1e), it has previously been shown to be due to both the genes falling under a shared open chromatin domain (Mutskov and Felsenfeld 2009).

Further, focusing on our primary aim, we also observed that the lncRNA *MALAT1* showed significant negative correlation with *SCYL1* in a large number of cancer types, consistent with a previously reported negative *cis*-regulatory effect on this gene (Zhang *et al.*, 2012) (Figure 1e). Similarly, *KCNQ1OT1* showed significant negative correlation with *KCNQ1* in several cancers, supporting its previously reported role in repressing *KCNQ1* (Du *et al.*, 2004) (Figure 1e). We here posit that, unlike positive correlation, such cases of negative correlation between a coding/lncRNA pair are more likely to be informative of putative *cis*-regulatory interactions.

### Detailed investigation of negatively correlated lncRNAs/coding pairs

We found in total 193 pairs of lncRNAs and coding genes (involving 178 unique lncRNAs and 188 unique coding genes) that showed negative expression correlation in the majority of cancers and that reached significance (*P <* 1e-5) in at least one cancer type. Here onwards we refer to these pairs as the lncRNAs/coding negatively correlated (LCN) set (Table S2). We similarly defined a coding/coding negatively correlated set (CCN, 1412 pairs) using the same criteria, as well analogous positively correlated sets (LCP, 4094 pairs; CCP, 23,824 pairs).

**Figure 2:**
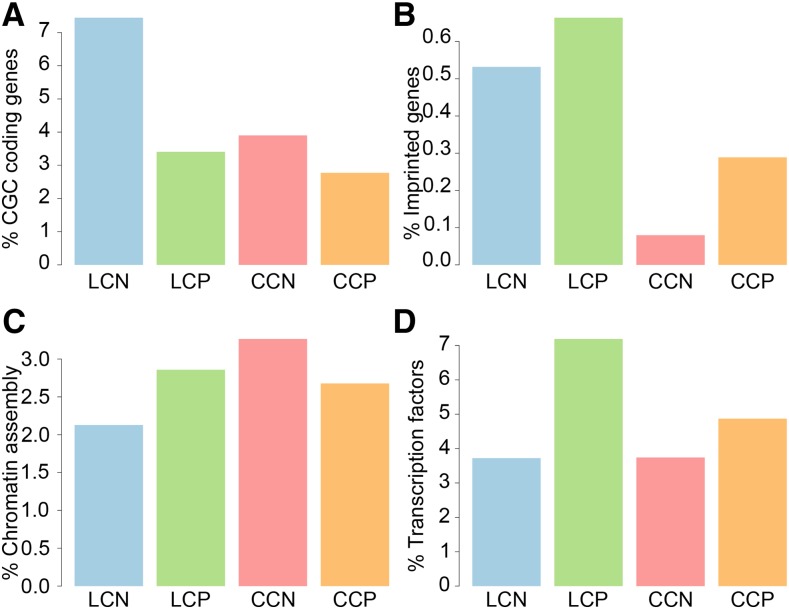
Enrichment of gene classes within specific subset of coding/lncRNA and coding/coding pairs showing positive or negative correlation in majority of cancer types A) Cancer Gene Census genes B) Imprinted genes C) Genes involved in chromatin assembly D) Transcription factors. LCN stands for lncRNA/coding negatively correlated, LCP is lncRNA/coding positively correlated set, CCN is coding/coding negatively correlated set and CCP is coding/coding positively correlated set.

To characterize the coding genes in the LCN set, we tested for enrichment of several different gene categories. Notably, we found that known cancer driver genes from the cancer gene census (Futreal *et al.*, 2004) were overrepresented in the LNC set compared to the other ones (fisher test *P* < 0.01, Figure 2a), while no enrichment was seen in this set for imprinted genes, chromatin modifiers or transcription factors (Figure 2b,c,d). 14 of these coding/lncRNA pairs in the LCN set, where the coding gene is reported to play a role in cancer are listed in Table 1. The cancer gene census enrichment suggests that cancer driver genes may be under the control of *cis*-regulatory lncRNAs to a larger extent than other genes, and the list of such lncRNAs/coding pairs thus constitutes a resource for selecting candidate lncRNAs with a functional role in cancer for experimental evaluation.

**Table 1:** Coding genes from the cancer gene census that show negative correlation of expression with a proximal lncRNA in majority of cancer types, with the correlation being significant (*P* < 1e-5) in at least one cancer. +ρ and -ρ indicates the number of cancer types that show significant positive or negative correlation, respectively, at the *P* < 1e-5 level. *Tau*C and *Tau*L give the *Tau* expression specificity score while PhastconC and PhastconL indicates the phastCons 100-way sequence conservation score for the coding gene and the lncRNA

Coding	Class	LncRNA	+ρ	-ρ	TauC	TauL	PhastconC	PhastconL
*BLM*	TSG	*CTD-3094K11.1*	1	2	0.68	0.70	0.49	0.02
*COL1A1*	Oncogene	*RP11-893F2.13*	0	1	0.86	0.97	0.51	0.05
*DICER1*	TSG	*DICER1-AS1*	0	3	0.42	0.65	0.65	0.07
*FBXO11*	—	*AC079807.2*	1	5	0.45	0.76	0.61	0.33
*KMT2A*	Oncogene	*RP11-770J1.3*	0	3	0.52	0.82	0.68	0.04
*LCP1*	—	*CPB2-AS1*	0	1	0.91	0.94	0.43	0.03
*NF1*	—	*RP11-848P1.5*	0	2	0.53	0.86	0.49	0.23
*NR4A3*	—	*RP11-60I3.5*	0	2	0.84	0.66	0.78	0.07
*RHOH*	—	*RP11-395I6.3*	2	5	0.95	0.80	0.15	0.09
*SDHC*	—	*RP11-122G18.8*	0	4	0.37	0.84	0.40	0.06
*SET*	—	*HMGA1P4*	0	1	0.47	0.99	0.60	0.13
*STAG2*	—	*RP1-315G1.3*	0	2	0.41	0.90	0.89	0.55
*STIL*	—	*RP1-18D14.7*	0	1	0.63	0.96	0.42	0.11
*TOP1*	—	*RP1-1J6.2*	0	2	0.47	0.93	0.93	0.09

### Further characterization of lncRNAs in negatively correlated lncRNAs/coding pairs

**Figure 3:**
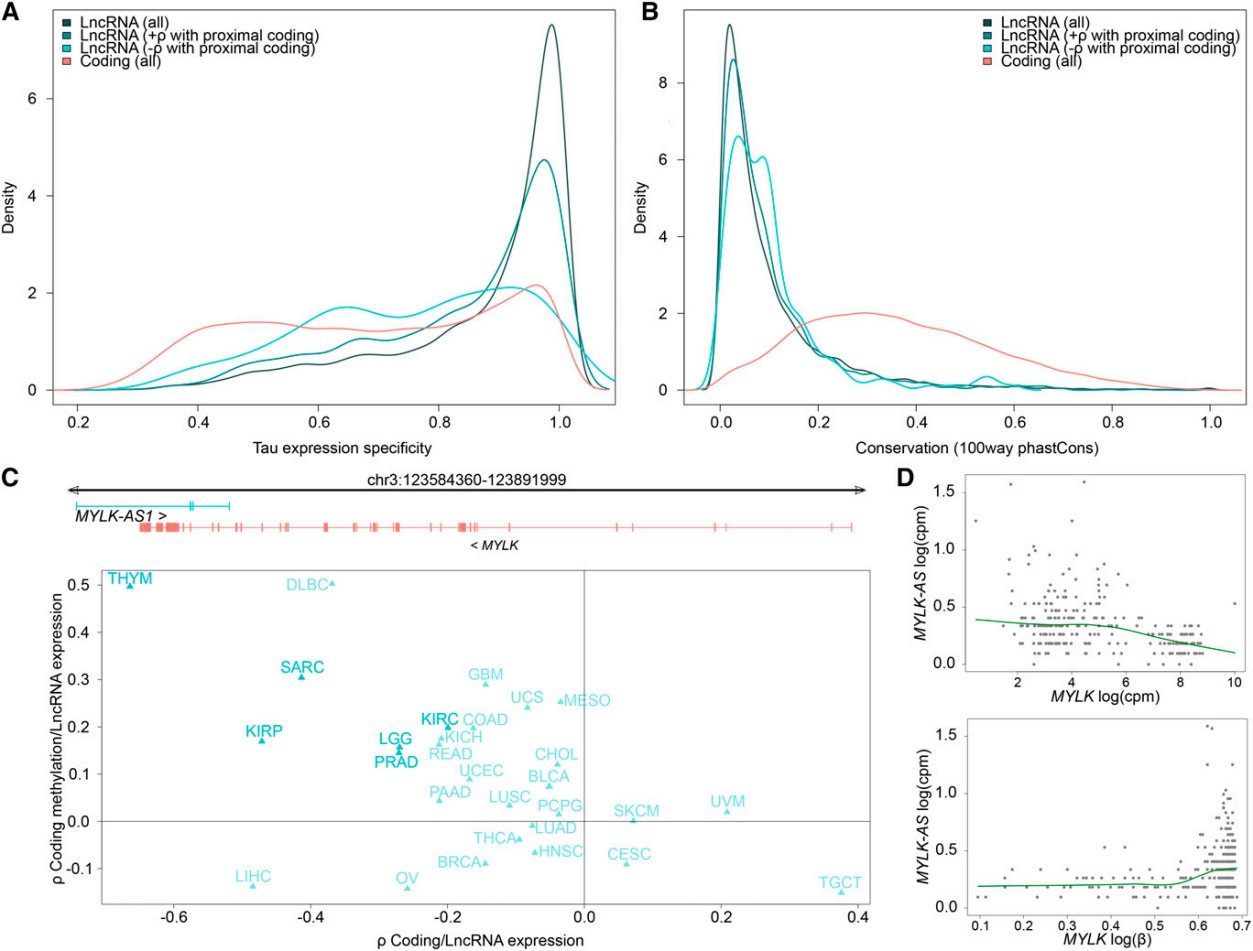
Expression specificity, sequence conservation and association of lncRNA expression and coding TSS methylation. A) Cancer type expression specificity distribution is able to demarcate lncRNAs negatively correlated with their proximal coding genes. B) A small subset of lncRNAs that shows negative correlation with their proximal coding genes show elevated levels of sequence conservation, indicative of a potential functional importance. C) Comparison of *MYLK-AS1* expression against *MYLK* gene expression and *MYLK* TSS methylation across multiple cancer types. Cancer types where the expression correlation and expression/methylation correlation are both significant are marked in bold. D) The relationship between *MYLK* expression, *MYLK-AS* expression and *MYLK* promoter methylation across 259 sarcoma samples. Log(cpm) represents gene expression while log(β) is for coding TSS methylation beta values. LncRNA (+ρ with proximal coding) is the LCP dataset and LncRNA (-ρ with proximal coding) is the LCN dataset.

We added further value to our predictions by considering metrics that may be indicative of gene function in the case of lncRNAs, like expression specificity, sequence conservation, nuclear localization, and association of lncRNA expression with methylation at coding gene promoters. A core tenet of lncRNA biology is their highly tissue specific expression pattern in comparison to coding genes (Cabili *et al.*, 2011; Derrien *et al.*, 2012), where a recent report has identified several tissue-specific lncRNAs that are also expressed precisely in certain cancer types (Iyer *et al.*, 2015). Thus we investigated the cancer type specificity of all lncRNAs and coding genes using the *Tau* specificity score, which ranges from 0 and 1 where 0 implies ubiquitous expression while 1 indicates a highly restricted expression pattern across a given set of samples (Yanai *et al.*, 2005). We found that lncRNAs tend to be expressed in a significantly more cancer type-specific manner compared to coding genes (Student’s *t*-test, *P* < 2.2e-16; Figure 3a). However, interestingly lncRNAs in the LCN set showed significantly lower specificity compared to the LCP set (Student’s *t*-test, *P =* 3.6e-11) and all lncRNAs (Student’s *t*-test, *P =* 1.6e-15). Presumably, being negative correlated with a coding gene implies a more complex, coding-like, expression pattern across cancers types.

Further, we characterized the LCN set in terms of evolutionary sequence conservation. Although lncRNAs generally show a low level of conservation, highly conserved blocks are reported within well-characterized lncRNAs like *XIST*, and these blocks sometimes coincide with functionally important sequence elements (Brown *et al.*, 1992; Fang *et al.*, 2015; Holding 2001) . Here we compared the level of sequence conservation in the candidate lncRNAs, since this metric may indicate a possible functional significance. Only conservation in exonic regions not overlapping coding exons was considered. Although LCN lncRNAs showed higher conservation on average, the difference was not statistically significant (Figure 3b, Student’s *t*-test *P* = 0.7). None the less, it is worth noting that we find isolated instances of high sequence conservation (average phastcons 100-way score > 0.5) for lncRNAs lying proximal to coding genes like *STAG2*, *KMT2E*, *ZNF720*, *ERBB2IP*, *FBXL18*, involved in chromosomal segregation, cell cycle progression, transcription regulation, cellular adhesion and apoptosis.

A prerequisite for *cis*-regulatory function is nuclear localization. Hence we used the Cytoplasmic/Nuclear Relative Concentration Index (RCI), available from the LncAtlas database (Mas-Ponte *et al.*, 2017), to investigate the predicted subcellular localization of lncRNAs in the LCN set across 15 human cell lines (Table S3). Interestingly, the RCI distribution was shifted toward nuclear localization in most cell lines (median below zero in 12/15 cases), and in comparison with all lncRNAs, RCI values were significantly lower in 12/15 cases (*P* < 0.05, Student’s *t*-test; Figure S6). RCI values were also lower compared to LCP lncRNAs in most cell lines (13/15 with 4/15 being significant at the *P* < 0.05 level; Figure S6). The LCN set thus shows a preference for nuclear localization, compatible with putative *cis*-regulatory roles.

Finally, we proceeded to investigate the relationship between lncRNA expression and methylation levels at coding transcriptional start sites (TSS). A classical mechanism of gene repression in *cis* by long non-coding RNAs is through methylation of the genomic neighborhood, and examples include *KCNQ1OT1* and *AIR* (Mancini-DiNardo *et al.*, 2006; Sleutels *et al.*, 2002). We checked the LCN dataset for potential candidates that may involve this mechanism, by comparing expression of the lncRNAs with the methylation levels at the transcription start site (TSS) of their coding counterparts in individual cancer types, arguing that a positive correlation between these variables might be indicative of repression through methylation (Table S4). Notably, a positive correlation (ρ > 0) between these variables was observed for 54% of the LCN set (97 unique lncRNAs and 93 unique coding genes), including the well-characterized *KCNQ1OT1*. An interesting case to highlight is the *Myosin light chain kinase* (*MYLK*) gene and its antisense lncRNA *MYLK-AS1*, where the coding/lncRNA pair show coupled association of expression and methylation in 19/32 cancer types (Figure 3c), with the strongest trend in sarcoma (Figure 3d; coding/lncRNA expression ρ = -0.41, *P* = 1.31e-11; coding methylation/lncRNA expression ρ = 0.30, *P* = 6.40e-6). *MYLK* is a well-known muscle differentiation marker gene that is associated with specific subtypes of sarcoma (Baird *et al.*, 2005) and leiomysarcoma (Beck *et al.*, 2009). Although more work would be needed to establish *MYLK-AS1* as an inducer of promoter methylation and negative regulator of *MYLK*, these observations exemplify how our correlative analysis can provide starting points for future experimental studies.

### Concluding remarks

Long non-coding RNAs can induce or repress the expression of neighboring coding genes through diverse mechanisms, including direct binding to DNA or RNA or by acting as scaffolds or tethers for chromatin modifiers. Although lncRNAs like *MALAT1* and *lincRNA-p21* are implicated in various cancers, we currently do not possess an in depth understanding of the role of lncRNAs in cancer initiation and development. A primary reason for this lies in the fact that even though well above 10,000 lncRNAs are reported to be present in the human genome (Derrien *et al.*, 2012), less than 100 are functionally well characterized.

Lately, the availability of large-scale genomics data pertaining to cancer genomes has provided us with immense resources for identification of novel lncRNAs with putative roles in cancer. Here we have analyzed an unprecedented number of transcriptomes, sourced from over 9000 primary tumor samples spread across 32 cancer types, to determine correlative relationships between lncRNAs and protein-coding neighbors, with the aim of identifying lncRNAs with potential *cis*-regulatory functions that may be relevant in cancer. We suggest that negative correlations between coding genes and their proximal lncRNAs may less likely arise due to trivial mechanisms such as shared active chromatin domains, and propose a set of 193 lncRNAs that are potential negative *cis*-regulators of nearby coding genes. Several of the identified lncRNAs lie proximal to coding genes implicated in the development of one or more cancer types. These results must be seen as predictions that can form the basis of further experimental investigations, and it should be noted that negative correlations might also arise for other reasons. For example, a recent study described two cases of inversely expressed lncRNAs/coding pairs that were independently regulated (Goyal *et al.*, 2017). Notably, information on expression specificity, sequence conservation and relationships between lncRNA expression and coding promoter methylation brings additional value to our compendium, which may aid in the discovery of novel *cis*-regulatory molecules with potential roles as therapeutic targets or biomarkers in cancer.
